# The critical role of emotional communication for motivated reasoning

**DOI:** 10.1038/s41598-024-81605-6

**Published:** 2024-12-30

**Authors:** Ingo Wolf, Tobias Schröder

**Affiliations:** 1 Research Institute for Sustainability – Helmholtz Centre Potsdam (RIFS), Berliner Straße 130, 14467 Potsdam, Germany; 2 Potsdam University of Applied Sciences, Kiepenheuerallee 5, 14469 Potsdam, Germany

**Keywords:** Neuroscience, Psychology

## Abstract

Persuasive appeals frequently prove ineffective or produce unintended outcomes, due to the presence of motivated reasoning. Using the example of electric cars adoption, this research delves into the impact of emotional content, message valence, and the coherence of pre-existing attitudes on biased information evaluation. By conducting a factorial survey (*N* = 480) and incorporating a computational model of attitude formation, we aim to gain a deeper insight into the cognitive-affective mechanisms driving motivated reasoning. Our experimental findings reveal that motivated reasoning is most pronounced when persuasive appeals employ a combination of emotional and rational elements within a negatively valenced argument. Furthermore, our computational model, which estimates belief and affect adjustments underlying attitude changes, elucidates how message framing influences cognitive-affective processes through emotional coherence. The results provide support for a negative correlation between shifts in coherence in response to new information and the propensity for motivated reasoning. The research contributes to computational models of opinion dynamics and social influence, offering a psychologically realistic framework for exploring the impact of individual reasoning on population-level dynamics, particularly in policy contexts, where it can enhance communication and informed policy discussions.

**Supplementary Information:**

The online version contains supplementary material available at 10.1038/s41598-024-81605-6.

## Introduction

The adoption of electric vehicles (EVs) is a critical component in reducing greenhouse gas emissions and transitioning to a low-carbon transportation system^1^. After several years of notable global growth in EV registrations, the rate has recently slowed or even declined in some countries. Research into the behavioral aspects of EV acceptance has identified various factors that influence and potentially hinder the adoption of alternative fuel vehicles, such as economic considerations, technological advancements, infrastructural development, and policy support and incentives^2–6^. Recent literature has also shown that people’s attitudes towards and adoption behavior of new vehicle technologies are substantially influenced by interpersonal communication and persuasion^7,8^. The interpersonal exchange of information about innovation attributes and (emotional) experiences can raise awareness, reduce uncertainty, and provide practical knowledge about the adoption process, thereby encouraging others to follow suit. However, it can also reinforce skepticism and hinder adoption if the shared information is negative or if influential individuals express doubts. Here, we focus on the question of under which cognitive-affective conditions receivers are persuaded by rational and emotional appeals about different vehicle types.

Whether interpersonal communication is persuasive depends on the source of communication, the message type and content and, on the psychosocial characteristics of the receiver. A large body of theoretical research has emerged, providing a detailed understanding of how persuasion works and why its effectiveness can vary across different situations and individuals^9–11^. More recent work has explored cognitive-motivational aspects of information processing in social communication, particularly the influence of motivated reasoning in persuasive communication^12,13^. Motivated reasoning refers to a cognitive process where directional (motivational) goals, such as wishes or preferences, drive individuals to form beliefs, interpret information, and make decisions that support their desired conclusions rather than objective evidence^14^. Emotional and affective processes play a critical role in motivated reasoning^15^. Affective-emotional states can enhance the salience of certain pieces of information, leading to biased assimilation and interpretation to maintain a coherent, yet biased, cognitive state^16–18^.

Numerous empirical studies on motivated reasoning provide substantial evidence across diverse domains that human information processing is both influenced and constrained by distinct directional goals such as maintaining pre-existing beliefs^19^ and attitudes^20,21^, affirmation of culturally ingrained values^22^, protecting social identities^23,24^ or religious worldviews^25^. People employ a range of strategies to achieve desired outcomes and resist persuasive appeals, including selectively avoiding information, discounting the source, and experiencing negative emotional reactions^26^. Furthermore, several studies have shown that prolonged exposure to counterattitudinal messages may even lead to a backfire effect—also referred to as backlash or boomerang effect—exacerbating attitudinal polarization among social groups with opposing views in both offline and online communication^27–29^. This psychological phenomenon occurs when attempts to persuade people to change actually strengthen their original position when the message challenges their core values, beliefs, and affective representations. The findings on the backfire effect are, however, mixed and inconsistent, with many studies failing to reliably replicate its occurrence^30^, highlighting critical limitations in existing theoretical frameworks. 

These gaps center on two important aspects. First, while numerous studies have examined this cognitive process in environmental contexts, particularly climate change^19,24,31–37^, these investigations have largely concentrated on broader, less personally relevant topics. Such research typically employs messages about scientific evidence that may seem disconnected from individuals’ daily lives and behaviors. Second, many studies do not systematically account for the affective processes induced by the (emotional) messages. While Bayesian principles can explain the integration of new evidence for ‘cold’ cognitive processes, this approach appears incompatible with Bayesian rational calculus principles when belief updating is driven by “hot” cognitions, such as strong directional motivations or emotional stakes^38^.

An alternative account of motivated reasoning phenomena comes from coherence-based reasoning frameworks^39–42^. Coherence-based reasoning frameworks, such as Paul Thagard’s theory of emotional coherence, are modeled using connectionist approaches that assert mental processes can be represented by brain-like neural networks.  Emotional coherence refers to the state in which a cognitive-affective network (i.e., cold and hot cognitive elements) achieves balance by ensuring that positively linked elements are similarly activated and negatively linked elements are dissimilarly activated. This balanced mental state is reached by adjusting the strength and configuration of nodes within the network to maximize consistency and satisfy constraints, leading to an overall state of emotional coherence. Unlike Bayesian updating, which relies on rational and probabilistic evidence integration, emotional coherence suggests that devaluing conflicting information while favoring or endorsing supporting data can enhance the coherence of existing mental representations, including goals, beliefs, and emotions. In these networks, each element of a mental task is represented as a symbolic interconnected node and processed through parallel constraint-satisfaction mechanisms (PCS) to maximize consistency and satisfy constraints^41,43–48^. PCS models, in general, are a formal conceptualization of cognitive consistency, drawing from established theories like cognitive dissonance^49^ and congruity theory^50^.

Existing PCS models of attitudes offer a practical, formalized framework for understanding the complex structures and processes involved in attitude change, along with a quantitative measure of coherence^42^. However, previous approaches, with some notable exceptions such as Simon et al.^51^, have mainly relied on aggregated empirical or simulated data, limiting their ability to provide detailed empirical insights at a cognitive-affective level. These models lack the capacity to generate testable predictions about specific mental structures that either encourage or discourage motivated reasoning based on the direction of advocacy (inconsistency or consistency of beliefs and affects) and the nature of the argumentation (rational or emotional) in persuasive communication. Furthermore, most coherence models have not explicitly considered the influence of affective processes associated with motivated reasoning, as documented in empirical studies of motivated reasoning^52^.

In this study, we integrate experimental data with computational modeling to explore and understand the cognitive and affective processes that underlie motivated reasoning in response to persuasive appeals. First, we conducted an online factorial survey experiment, allowing us to analyze whether attitudinal responses to persuasive appeals differ across various levels and valences of emotional message frames. We aimed to test the hypothesis that motivated reasoning contributes to a negative evaluation of information sources and leads to attitude polarization. Second, we simulated the process of information exchange and integration by creating a parallel constraint satisfaction network (PCS) model for each sender (vignette statement) and receiver (participants) in the experiment. PCS models represent issue-specific mental structures as nodes (goals and attitude objects) and connected links (beliefs and affective meanings). For each of the three experimental conditions, we estimated the adjustments made by respondents to their pre-existing network structures based on empirically reported attitude changes. This allowed us to identify specific mental structures and conditions that are most susceptible to motivated reasoning. Furthermore, our approach allowed us to quantitatively assess the theoretically predicted increase in overall coherence resulting from motivated reasoning.

## Factorial survey

### Methods

We conducted a factorial survey experiment with statements both advocating for and opposing the use of internal combustion engine (ICE-cars) and electric cars (E-cars). By using a vignette design, we aimed to investigate the interplay between pro- and counterattitudinal information, emotional content, and the presence of motivated reasoning. The vignettes described the attitudes and experiences of a hypothetical person advocating for or against ICE- or E-cars under three experimental conditions: rational, emotional, and a combination of emotional and rational argumentation.

The persuasive messages presented to participants were selected from a broader set of vignettes that varied along four dimensions, each featuring two to five distinct characteristics (levels) as outlined in Table A1. These dimensions included: (1) persuasive communication type (alligned with experimental conditions), (2) the vehicle powertrain (conventional internal combustion engine (ICE) vs. electric (E-) cars), (3) beliefs about how well different vehicle types fulfill transportation-related goals (personal mobility independence, travel comfort, eco-friendliness, driving pleasure & experience, and environmental impact concerns), and (4) attitudinal valence (positive or negative) of the attitude toward the vehicle. While the dimensions were consistent across vignettes, the characteristics were systematically combined. To enhance ecological validity while maintaining diversity of argumentation, we combined statements about the vehicles’ perceived contributions to these transportation goals into pairs, with both statements sharing the same valence (either both positive or both negative). This pairing strategy yielded 10 distinct combinations (e.g., good for independence and comfort in positive vignettes, or poor for eco-friendliness and limited mobility independence in negative ones), trading off the coverage of goal-related belief combinations with vignette length and cognitive load.

There were 120 unique vignettes overall, which we organized into 12 balanced sets with four sets per experimental condition (40 vignettes/condition). Each set contained 10 vignettes (five ICE, five E-car), with balanced positive and negative valence statements across vehicle types within each condition. Complete vignette examples and detailed condition specifications are provided in the Supplementary Material.

#### Pilot study

To ensure the specificity and relevance of the characteristics of the three message types, all vignettes underwent pilot testing. Participants in the pilot study (*n* = 180) were recruited and incentivized through a commercial online research panel in Germany, namely Bilendi & respondi Group. They were randomly assigned to one of three groups, each consisting of 40 vignettes of a specific type (i.e., rational, emotional, or combined). We excluded five individuals due to incomplete data, resulting in a final sample of *n* = 175 respondents (with 56 in the rational messages group, 59 in the emotional messages group, and 60 in the combined messages group). In the study, participants rated the emotionality and information content of each vignette on a six-point scale, ranging from ‘not at all’ (1) to ‘completely’ (6). As anticipated, participants considered the rational messages significantly more informative than the combined statements and the emotional ones (rational: M = 3.58, SD = 0.09; combination: M = 3.19, SD = 0.08; emotional: M = 3.06, SD = 0.08; F(2,172) = 9.79, *p* < 0.0001). Additionally, they evaluated emotionally framed messages as having significantly more emotional content than combined and rational statements (emotional: M = 4.23, SD = 0.10; combined: M = 4.13, SD = 0.10; rational: M = 3.80, SD = 0.1; F(2,172) = 5.36, *p* < 0.005).

#### Main study

In the main study, we implemented a 3 × 2 × 10 × 2 mixed factorial design to examine responses to various persuasive messages about the use of internal combustion engine (ICE) cars and electric cars (E-cars). The between-subjects factor was the type of persuasive communication: rational, emotional, or combined. The within-subjects factors included the vehicle type, the general valence of the message, and beliefs about the vehicle’s contribution to domain-specific goals. Our sample consisted of 480 participants, with a gender distribution of 47.1%, aged between 18 and 65 years, and a mean age of 46.63 (SD = 13.90). These participants were also recruited by Bilendi & respondi Group. All participants held a valid driver’s license, and the majority (89.8%) indicated having at least moderate knowledge of electric vehicles.

Participants were randomly allocated to one of three experimental conditions, with each group consisting of 160 participants: (i) *rational* persuasion, which featured vignettes presenting factual and goal-oriented arguments; (ii) *emotional* persuasion, where vignettes conveyed emotional experiences with the different car types; and (iii) *combined* persuasion, involving vignettes that integrated both goal-oriented and emotional arguments. Each participant received one set of 10 vignettes, which was randomly selected from four sets available for each condition. Additionally, the order of vignettes was randomized for each participant to avoid order effects.

Before presenting the vignettes, we assessed participants’ pre-existing attitudes toward conventional internal combustion engine (ICE-cars) and electric cars (E-cars). For ICE-cars, we used 13 items, and for E-cars, we used 15 items. Participants were asked to rate their agreement or disagreement on a six-point Likert scale in response to statements related to various goal-related aspects of transport mode use (e.g., cost efficiency goal: ‘the cost of driving an E-Car is too expensive for me’; independence goal: ‘Driving an ICE-car means freedom to me’). The items for each transport mode were aggregated into corresponding ICE-car and E-car attitude scales, by taking the mean of the ratings and rescaled to a range of −1 to + 1. To gather the necessary empirical parameters for the PCS model described later, participants were requested to indicate the importance of five goals related to their choices of transport modes (independence, comfort, eco-friendliness, driving experience, good conscience) on a six-point Likert scale. They were also asked to rate the emotional valence of these goals and the transport mode options (ICE-cars and E-Cars) on a semantic differential scale, which ranged from − 4 to + 4. Furthermore, participants provided their beliefs regarding the extent to which these goals could be satisfied by using ICE-cars and E-Cars on a six-point Likert scale.

In the main segment of the factorial survey, the set of 10 vignettes were displayed on separate screens . After each vignette, four dependent variables were assessed. Participants were required to provide responses on the following: (a) Their level of agreement with the statements, rated on a scale from 1 (strongly disagree) to 6 (strongly agree), to measure their cognitive dissonance with the messages, (b) the likability of the author, evaluated on a scale from 1 (very unpleasant) to 6 (very likable), aimed at quantifying the extent to which they discounted the source of the messages, (c) the competence of the author, rated on a scale from 1 (very low) to 6 (very high), also employed to gauge the discounting of the message source and (d) whether they perceived an attitude change in a more positive or negative direction after evaluating the persuasive message, assessed on a seven-point scale ranging from ‘much more negative’ (1) to ‘much more positive’ (7).

All procedures conducted in this study adhere to the ethical principles outlined in the 1964 Helsinki Declaration and its subsequent revisions, or equivalent ethical standards. The data collection followed the institutional guidelines of ESOMAR21, as well as the international quality standard ISO 20252:2019. All study participants provided their informed consent to participate, with no impact on their physical or psychological well-being, privacy, or personal rights. The compliance with national regulations governing the protection of human subjects was reviewed and approved by the Ethics Committee of the Research Institute for Sustainability—Helmholtz Centre Potsdam.

## Survey results

We initiated our analysis by examining whether the pre-treatment attitudes toward internal combustion engine cars (ICE-cars) and electric cars (E-cars) varied among the experimental groups. A one-way ANOVA revealed no significant differences between the groups for both attitude objects (ICE-cars: F(1,318) = 0.0, *p* = 0.965; E-cars: F(1,318) = 0.28, *p* = 0.600). Therefore, we can confidently assume that the subsequent results are not influenced by disparities in prior attitudes across the groups.

**Fig. 1:**
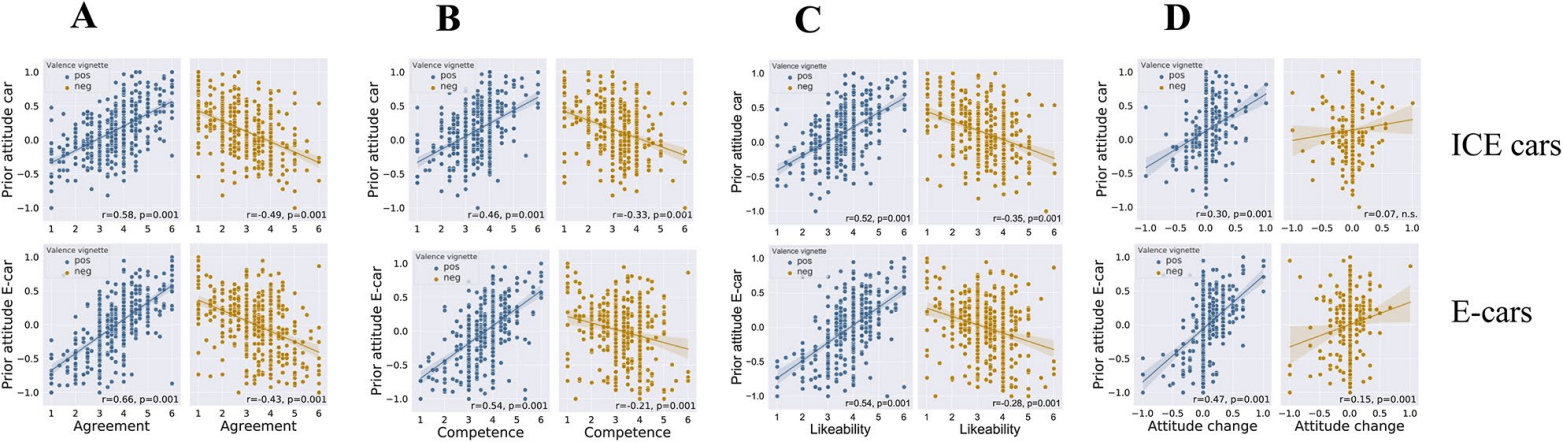
Correlations between prior attitudes towards conventional (upper panels) and electric cars (lower panels) and all vignette responses across conditions. Responses to positively valenced statements are plotted in blue and negatively valenced statements in gold. The solid lines are the correlation lines and shaded areas indicate a 95% CI. (**A**) Prior attitudes correlated with agreement to statements. (**B**) Prior attitudes correlated with the perceived competence of the sender of statements. (**C**) Prior attitudes correlated with the likeability of the sender. (**D**) Prior attitudes correlated with the self-reported attitude change.

To investigate the influence of prior attitudes on the assessment of information sources and subjectively perceived attitude changes, we utilized Pearson correlations. In Fig. 1A–D, we present separate correlations between prior attitudes toward conventional and electric cars and each vignette’s ratings, categorized into positively and negatively framed messages. It’s important to note that these analyses are aggregated across the three message types. The level of agreement with the statements displayed a strong association with participants’ prior attitudes (positive: ICE-car: *r* = 0.58, *p* = 0.001; E-car: *r* = 0.66, *p* = 0.001; negative: ICE-car: *r* = 0.49, *p* = 0.001; E-car: *r* = 0.43, *p* = 0.001). The results underscore the presence of motivated reasoning across both transportation modes and message framings (positive/negative). Messages aligning with participants’ pre-existing attitudes led to authors being perceived as more likable and competent than those whose messages significantly diverged from these attitudes. Although perceived attitude changes correlated with respondents’ prior beliefs, this correlation was less pronounced (Fig. 1D). Positive appeals reinforced attitudes in individuals with strong positive pre-existing attitudes (ICE-car: *r* = 0.30, *p* = 0.001; E-car: *r* = 0.47, *p* = 0.001). In contrast, negative messages concerning ICE-cars did not yield significant results (*r* = 0.07, p = not significant), and demonstrated a weak relationship for E-cars (*r* = 0.15, *p* = 0.01). Notably, a backfire effect occurred, wherein attitudes shifted in the opposite direction of the intended persuasive appeals among respondents with prior negative attitudes towards these transportation modes under the positive condition, and likewise for those with pre-existing positive beliefs in the negative condition. Consequently, they indicated even more pronounced positive attitudes after exposure to the message.

Overall, the correlations between prior attitudes and message evaluations were more pronounced for positive statements than for negative ones. This pattern was consistent for positively valenced messages about E-cars compared to ICE-cars, with a contrasting tendency observed for negative statements about the two vehicle types.

**Fig. 2:**
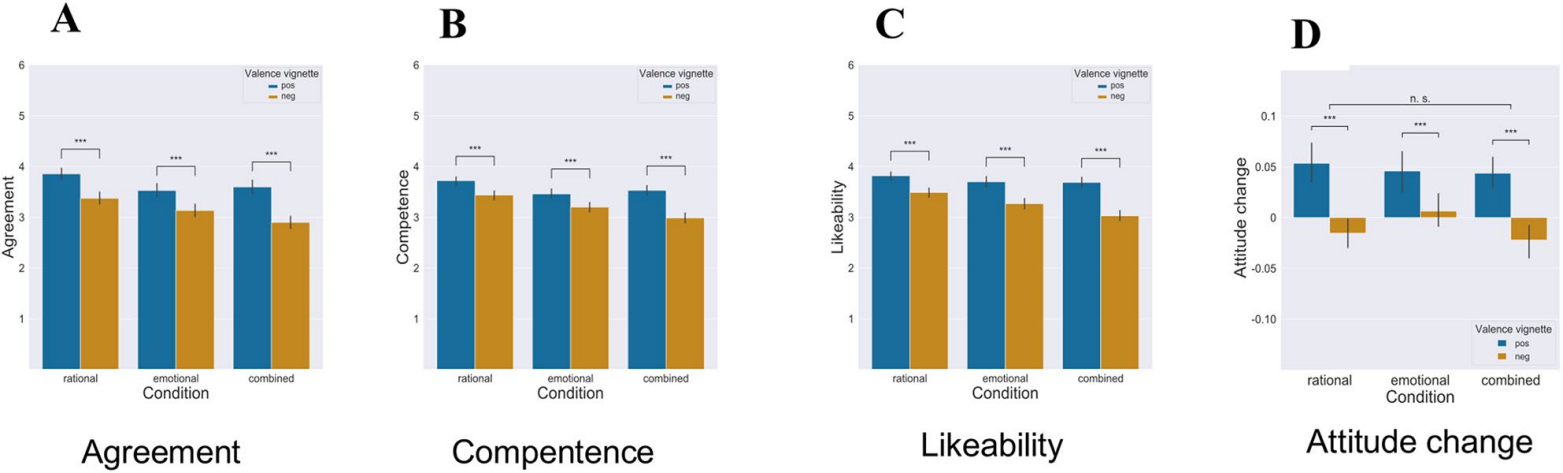
Mean ratings on all vignette responses across conditions, by experimental condition and valence of statements (blue: positive valence; gold: negative valence). Error bars represent standard error of the mean. (**A**) Agreements to vignettes. (**B**) Perceived competence of sender. (**C**) Likeability of sender. (**D**) Self-reported attitude change.

Subsequently, we conducted a separate analysis of the effects of positive and negative persuasive messages. In Fig. 2A–D, we present the average responses to all vignette-related measures, categorized by experimental condition and valence. Consistent with the notion that positive messages tend to be more appealing and persuasive than negatively valenced ones^53^, participants’ ratings, on average, were significantly higher for positive information. Separate dependent *t*-tests revealed substantial differences across all three conditions concerning: *agreement* with the statements (rational: t(159) = 6.50, *p* < 0.001; emotional: t(159) = 4.56, *p* < 0.001; combined: t(159) = 6.88, *p* < 0.001), perceived *competence* of the sender (rational: t(159) = 5.88, *p* < 0.001; emotional: t(159) = 4.83, *p* < 0.001; combined: t(159) = 7.76, *p* < 0.001), likability of the sender (rational: t(159) = 6.06, *p* < 0.001; emotional: t(159) = 6.96, *p* < 0.001; combined: t(159) = 8.69, *p* < 0.001), and *perceived attitude shift* in response to the information (rational: t(159) = 5.69, *p* < 0.001; emotional: t(159) = 2.60, *p* < 0.01; combined: t(159) = 4.24, *p* < 0.001).

It is important to note that despite the generally higher appeal of positive messages, our findings suggest a nuanced relationship between positive information and attitude changes. The observed backfire effect underscores the complexity of persuasive messaging, particularly when it conflicts with pre-existing beliefs. Positive messages may not always result in the intended attitude change, especially among individuals with strongly held beliefs. This highlights the need for a deeper understanding of the interplay between message valence and prior attitudes in shaping responses to persuasive appeals.

attitude change.

In our analysis of the impact of different types of persuasive appeals on perceived attitude changes, as shown in Fig. 2D, we conducted a one-way ANOVA with the type of persuasive appeal (rational, emotional, combined) as a between-subjects factor. This was followed by Tukey’s HSD post-hoc tests (*p* < 0.05). Surprisingly, we observed no significant differences either among the groups or in any pairwise comparisons between groups, whether for positive or negative messages (F(2,477) = 0.15, *p* = 0.8590, η_*p*_^2^ = 0.0003), nor for negative messages alone (F(2,477) = 1.80, *p* = 0.166, η_*p*_^2^ = 0.0006). Conversely, when assessing evaluations regarding sender characteristics, we detected notable effects across all types of persuasive appeals (rational, emotional, combined). For rational statements, participants indicated the highest levels of agreement (positive: F(2,477) = 8.85, *p* = 0.001; negative: F(2,477) = 13.45, *p* = 0.001), sender competence (positive: F(2,477) = 7.23, *p* = 0.0001, η_*p*_^2^ = 0.029; negative: F(2,477) = 19.40, *p* = 0.001, η_*p*_^2^ = 0.075), and likability (positive: F(2,477) = 2.36, *p* = 0.10, η_*p*_^2^ = 0.009; negative: F(2,477) = 20.04, *p* = 0.001, η_*p*_^2^ = 0.077), regardless of whether the message was positively or negatively framed. Responses to emotional information were slightly, though not significantly, more positive than those to the combined condition, as indicated by the post-hoc test.

Drawing on emotional coherence theory^40^, which predicts that message effectiveness should systematically vary with recipient cognitive and affective characteristics, we conducted a detailed analysis of covariance (ANCOVA), controlling for perceived dissonance (i.e., agreement), prior attitudes, and their two-way interactions with the treatment. The main effect of message type on perceived attitude change showed slight significance (positive: F(2,477) = 3.29, *p* = 0.0379, η_*p*_^2^ = 0.014; negative: F(2,477) = 3.80, *p* = 0.0231, η_*p*_^2^ = 0.016). Notably, covariates, such as prior attitude towards E-cars (positive: F(1,478) = 9.32, *p* = 0.0024, η_*p*_^2^ = 0.019; negative: F(1,478) = 11.34, *p* = 0.0008, η_*p*_^2^ = 0.019), and agreement with the statement, had a significant effect on perceived attitude change (positive: F(1,478) = 215.83, *p* = 0.0001, η_*p*_^2^ = 0.312; negative: F(1,478) = 45.23, *p* = 0.0001, η_*p*_^2^ = 0.087). However, prior attitudes toward ICE-cars showed no significant influence on perceived attitude change (positive: F(1,478) = 0.76, *p* = 0.7829, η_*p*_^2^ = 0.018; negative: F(1,478) = 0.93, *p* = 0.3352, η_*p*_^2^ = 0.017). The results revealed a small yet significant interaction between message type and agreement for negatively framed statements (F(2,477) = 3.80, *p* = 0.0023, η_*p*_^2^ = 0.018), and a non-significant interaction for positively valenced statements (F(2,477) = 2.15, *p* = 0.117, η_*p*_^2^ = 0.009.

The theory of emotional coherence offers an explanation for this valence asymmetry: For negative messages, disagreement creates stronger cognitive conflict requiring more intensive resolution, making the pathways to coherence (i.e., message type) more critical. In contrast, positive messages may require less intensive coherence-maximizing processes even under disagreement, as positive information generally requires less cognitive elaboration to integrate^54^. Under conditions of high dissonance, participants perceived combined messages as the most persuasive, suggesting that multiple pathways for establishing coherence are particularly effective when cognitive conflict is high. The dual nature of these messages helps reduce cognitive dissonance by offering both logical arguments and emotional appeal, making the information persuasive. When respondents showed higher agreement (lower dissonance) with the statements, both rational and emotional messages led to stronger perceived attitude changes. In these low-conflict situations, the cognitive system requires less intensive coherence-maximizing processes—rational messages can elicit changes through logical consistency while emotional messages achieve this by eliciting positive emotional responses.

A second ANCOVA examining the perceived competence of the message sender, with prior attitudes, agreement, and their interactions as covariates, showed that the effect of message type became non-significant for positively framed statements (F(2,477) = 0.58, *p* = 0.5595, η_*p*_^2^ = 0.002). However, it remained significant for negatively framed statements (F(2,477) = 5.12, *p* = 0.0063, η_*p*_^2^ = 0.021), with rational messages leading to the highest perceived competence. No significant interaction between message type and agreement was found, but the main effect of agreement was strongly associated with attributed competence (positive: F(1,478) = 680.07, *p* = 0.0001, η_*p*_^2^ = 0.588; negative: F(1,478) = 807.27, *p* = 0.0001, η_*p*_^2^ = 0.629). Additionally, prior attitudes towards E-cars significantly influenced competence attribution for negatively framed messages (positive: F(1,478) = 0.39, *p* = 0.5278, η_*p*_^2^ = 0.001; negative: F(1,478) = 25.12, *p* = 0.0001, η_*p*_^2^ = 0.050). This finding aligns with previous research indicating the impact of motivated reasoning on expertise perception and suggests that competence attribution to the sender was primarily driven by perceived dissonance with persuasive appeals rather than the type of argumentation or prior attitudes.

In summary, the empirical results demonstrate the influence of prior attitudes and dissonance on information evaluation, providing support for the proposed motivated reasoning processes. This effect was most pronounced for negatively valenced combined statements, resulting in more negative judgments about senders’ characteristics and lower persuasiveness compared to positive rational or emotional messages. The impact of different message types was primarily moderated by the level of dissonance or disagreement with the statements.

## Parallel constraint satisfaction model of motivated reasoning

In our experiment, we observed the process of motivated reasoning, wherein respondents tended to evaluate both the messages and the sender of the information in a way that aligned with their existing attitudes. To deepen our understanding of the underlying cognitive-affective structures and mechanisms that facilitate this process, we conducted an in-depth analysis by developing a computational model of attitude formation and change based on the parallel constraint satisfaction (PCS) theory of emotional coherence^40^.

PCS modeling plays a critical role in our study, offering multiple valuable insights. First, the parameters used in the simulation are derived from the empirical data collected during the study, reflecting real-world cognitive and affective dynamics as respondents interact with different types of messages. By employing these data-driven parameters, the PCS model translates complex mental tasks into a parsimonious and quantifiable computational framework of motivated reasoning. Second, while our experimental setup captures the outcomes, the simulation provides deeper insights into the underlying cognitive and affective processes driving motivated reasoning. It helps us understand how different types of information—emotional, rational, or combined—are processed by individuals, whether the information aligns with or contradicts their existing beliefs and feelings. This understanding is crucial for demonstrating how emotional and cognitive components interact to drive changes in attitudes, which might not be directly observable in the experimental data.

Furthermore, the PCS model allows us to quantify how adjustments in beliefs and affect under different conditions of message framing lead to changes in attitudes. By calculating the overall coherence within the network of beliefs and affects as they adjust in response to new information, the model helps in understanding how coherence (or lack thereof) in mental representations influences the persuasiveness of messages and the likelihood of attitude polarization. Finally, by validating the model’s assumptions and mechanisms through simulation, we enhance the generalizability of our findings. The model can be adapted and applied to other scenarios and populations, providing invaluable insights for future research exploring similar phenomena in different contexts. Thus, while the PCS model corroborates our empirical observations, it also extends our understanding by offering a detailed account of the processes that drive attitude formation and change, thereby providing a comprehensive framework for understanding motivated reasoning.

### Model structure

**Fig. 3:**
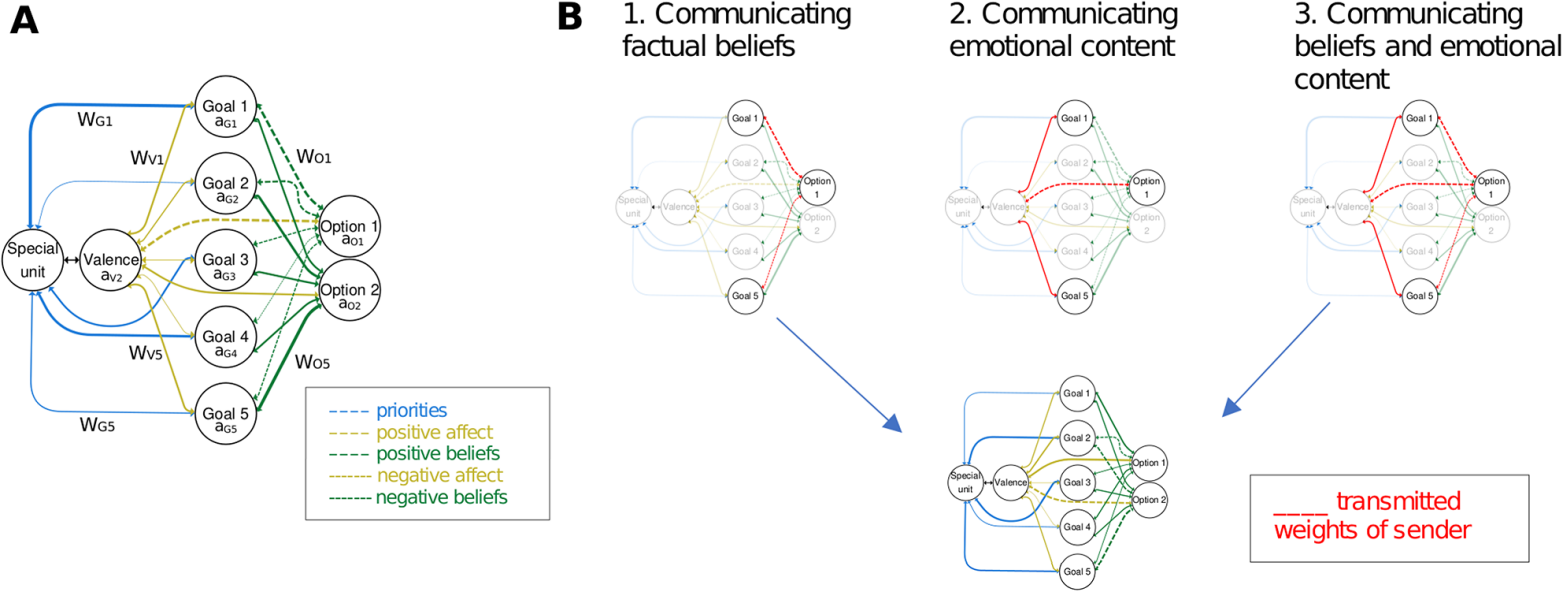
Parallel constraint satisfaction model (PCS) of attitude formation and change. (**A**) General PCS model of attitudes towards transport modes. Nodes in the network symbolize (from left to right), beyond a special unit for activating the spreading algorithm, the valence existing concepts, personal goals and the options of action in a mobility context. Links between the special unit and goal nodes (shown in blue) represent the priority/importance of each goal. Connections between the valence, goal and option nodes represent the affective association of concepts. Positive feelings are shown by solid lines and negative ones by dashed lines. Connections between goal and option nodes represent beliefs. Positive beliefs are displayed by solid lines and negative ones by dashed lines. All link weights are determined by the empirical ratings of participants in vignette experiment, described above in the main text. (**B**) Model of information transmission in vignette experiment for each experimental condition. The upper networks represent formal translations of vignette content into network structures (sender networks). These specify whichconnection weights should be modified in the receiver network (lower network) based on experimental condition: In the rational condition (1) only belief-related connection weights, in the emotional condition (2) only affect-related weights of affective associations, and in the combined condition (3) both types of weights are transmitted from sender to receiver networks. Red lines indicate the specific weights that are transmitted in each condition. The receiver network represents participants’ attitude structure and is modified according to the transmitted weights.

We designed the experimental framework as a one-way communication between two PCS models, each serving as a sender (i.e., the vignette statement) and a receiver (i.e., the respondent). Both models had identical network structures. The process involved four key steps, visually depicted in Fig. 3. Our starting point is a PCS network that represents the attitudes of the respondents (see Fig. 3A). Drawing from the general PCS model structure known as HOTCO (an abbreviation for “HOT Coherence”) as proposed by Thagard^29^, our computational model encompassed three interconnected components of attitudes (cf^41^) : cognitive (pertaining to beliefs about the relationship between goals and action options), affective (related to feelings about goals and action options), and conative (concerning behavioral tendencies regarding action options). Following HOTCO’s theoretical architecture, we explicitly separate cognitive and affective processing through distinct network structures. This separation is crucial as it allows us to model how emotional associations, and cognitive beliefs may independently influence attitude formation and change during persuasive communication.

In localist models like PCS networks, psychological concepts (e.g., goals or actions) are represented as individual units, and the links connecting these units denote the relationships between the concepts (e.g., beliefs or feelings). Our model, illustrated in Fig. 3A, features a central activation node (Special unit) and three interconnected layers of nodes. The Special unit serves two distinct functions: it acts as the source of activation for the network and enables individual differences in goal importance through priority links. The three layers comprise valence nodes that represent the pleasantness or unpleasantness of concepts, goal nodes that indicate the motives behind choices in transport modes (e.g. travelling eco-friendly), and option nodes representing the available transport mode choices. This architecture allows us to model the complex interplay between affective reactions, personal goals, and behavioral options in attitude formation.

Mutual constraints between these concepts were expressed through bidirectional links between nodes within each layer. Connections between goal and option nodes could either be excitatory (reflecting supportive beliefs, e.g., the goal of personal comfort aligns with the use of an E-car) or inhibitory (indicating conflicting beliefs, e.g., the goal of environmentally responsible travel behavior doesn’t align with the use of an ICE-car). Unlike some PCS implementations where options are mutually exclusive, we did not implement negative interconnections between transport mode options. This design choice reflects our theoretical position that attitudes toward electric and conventional vehicles aren’t necessarily antagonistic—individuals can simultaneously hold positive evaluations of both options’ different advantages.

Links between the valence node and goal or option units signaled positive (excitatory) or negative (inhibitory) affective associations with concepts. The strength of beliefs or affect was quantified by connection weights ranging from − 1.0 to 1.0. Lines connecting the general activation node and the goal units represented the personal importance of a goal, with weights ranging from 0 to 1.0. In this structure, each PCS network of the participants consisted of five goal nodes (independence, comfort, eco-friendliness, driving experience, good conscience) and two option nodes, representing attitudes towards the use of ICE- and E-cars. Based on this framework, we modeled the individual attitudes of each participant (*n* = 480) as a unique PCS model. These models were parameterized using the participants’ survey responses, which included information about the importance of goals, beliefs, and affective meanings of concepts (as described in the Factorial Survey section). All other model parameters were consistent with our prior studies (cf^42^).

An iterative updating algorithm simulates the process of attitude formation by spreading activations through the network, aiming to maximize coherence while considering all constraints within the given network structure^55^. At the initial time, t = 0, all nodes start with an activation close to zero, except for the general activation node, which is set to 1.0. The units’ activations are updated iteratively and in parallel until they reach a stable pattern of activation, with the degree of change falling below a predefined threshold. This updating process follows the equation:

1 $${a_j}\left( {t+1} \right)=\begin{array}{*{20}{c}} {{a_j}\left( t \right)\left( {1 - d} \right)+\left\{ {ne{t_j}\left[ {\hbox{max} - {a_j}\left( t \right)} \right]} \right\}if\,ne{t_j}>0} \\ {\left\{ {ne{t_j}\left[ {{a_j}\left( t \right) - \hbox{min} } \right]} \right\}if\,ne{t_j} \leqslant 0} \end{array}$$

where *a*_j_(t) is the activation of the unit *j* at iteration *t*. The constant parameter *d* (0.05) is the rate of decay of activation for each unit at every cycle, *min* is the minimum activation (-1.0) and *max* is the maximum activation (+ 1.0). The incoming activation from other nodes is computed by the connection weight *w*_*ij*_ between each unit *i* and *j*, the net input *net*_*j*_ to a node

2 $$ne{t_j}={\Sigma _i}{w_{ij}}{a_i}\left( t \right).$$

Updating continues until a stable level of activation is achieved, typically requiring fewer than 100 iterations. The ultimate activation of option nodes reflects the evaluation of an object, with higher positive values indicating a more positive attitude and higher negative values suggesting a more negative attitude. At this stage, the network’s overall coherence, represented as ‘Energy’^44,45^ is calculated by:

3 $$Energy\left( t \right)= - \sum\limits_{i} {\sum\limits_{j} {{w_{ij}}{a_i}{a_j}} }$$

Where *w*_*ij*_ is the weight between each unit *i* and *j*, and *a*_*i*_ (t) is the activation of unit *i* at time (*t*). This equation operates as follows: when the product of activation for two units aligns with the constraint between them, the network’s energy decreases; conversely, when the product of activation for two nodes contradicts the constraint between them, the network’s energy increases (see Fig. 2c). The network’s overall coherence is high when all cognitive and affective elements associated with one option align, while they do not align with the second option. Conversely, when beliefs and affect linked to both options are equally inconsistent, meaning that both option units are connected to a similar number of excitatory and inhibitory weights, attitudinal ambivalence arises, making it challenging to favor one option and subsequently reducing coherence.

In a second step, we created sender networks to formally represent the persuasive messages contained in the vignettes. These sender networks serve as a crucial intermediate step that translates verbal vignette content into precise network representations. For each vignette, we encoded its content as a specific pattern of connection weights in a network structurally identical to the receiver networks. For instance, a persuasive appeal promoting E-cars (*o*_*1*_) that emphasized environmental friendliness (*g*_*1*_) and comfort (*g*_*2*_) was represented by setting the corresponding connection weights (w_*g1−o1*,_ w_*g2−o1*_) and associated valence connections (w_*v−g1*,_ w_*v−g2*,_ w_*v−o1*_) to + 1.0, while all other weights were set to 0.01. Unlike receiver networks, sender networks only serve to specify weight patterns - their activation dynamics are not relevant for the subsequent information exchange process.

In the third step, we implemented the experimental conditions as different patterns of weight transmission between sender and receiver networks (see Fig. 3B). In the rational condition, only belief-related weights (connecting goals to options) were transmitted. In the emotional condition, only affect-related weights (connecting valence to goals and options) were conveyed. The combined condition transmitted both types of weights. In line with the experimental procedure, we generated simulations for 10 unique persuasive attempts per receiving agent, resulting in a total of 4800 (1600 per condition) simulated vignette treatments.

In the last step, we modeled the adaptation of attitudes resulting from persuasive communication attempts as modifications of connection weights within the receivers’ network structure. Drawing from the literature from network learning^56,57^, we developed an extended error-correction learning rule that captures how people maintain consistency among their beliefs when processing persuasive messages. Unlike previous learning processes in PCS networks^58^, these weight adjustments were specific, meaning that only links transmitted by the sender in the respective conversation and condition were altered in the receivers’ networks. For instance, in a rational persuasive communication regarding the use of electric cars that featured positive arguments about comfort and eco-friendliness, only the connections between the goal units (comfort and eco-friendliness) and the option unit (E-car) were modified.

The weight adjustment process is formalized through a polynomial function that captures key cognitive–motivational mechanisms of information processing^14,40^:

4 $$\Delta w\,=\,{b_1}\Delta {w_{s,r}}+{\text{ }}{b_2}{w_r}+{\text{ }}{b_3}\Delta {w_{s,r}}{w_r}+{\text{ }}{b_4}{w_r}^{2}+{\text{ }}{b_5}\Delta {w_{s,r}}^{2}$$

where *∆w*_*s, r*_ is the difference (i.e. dissonance) between the corresponding link weights of sender and receiver, i.e. the difference between existing beliefs and affects and new information, and and w_r_ represents the receiver’s existing attitude (connection weight before communication). The function also includes the interaction between *∆w*_*s, r*_ and *w*_*r*_ and is approximated up to quadratic order. Best fitting parameters (*b*_*1 − 5*_) were estimated by iterating systematically through the set free parameters in the permitted range between − 1.0 and + 1.0.

To identify optimal parameter values, we employed backpropagation as an optimization technique to minimize the total sum squared error function D across all vignette treatments, defined as:

5 $$D=\frac{1}{N}\sum\nolimits_{i}^{N} {{{\left( {{A_{sim}} - {A_{emp}}} \right)}^2}}$$

where *A*_*sim*_ represents the activation of the respective option unit in response to new information i.e. receivers simulated attitude towards a transport mode. *A*_*emp*_ is the empirically reported attitude after vignette treatment, calculated as by respondents’ original attitude plus the self-reported attitude change, which has been rescaled to values ranging between − 1 and + 1. N is the total number of vignette-participant pairs. The minimization of the error function is achieved by fitting free parameters (b1-b5) to the Eq. (4).

## Simulation results

**Fig. 4:**
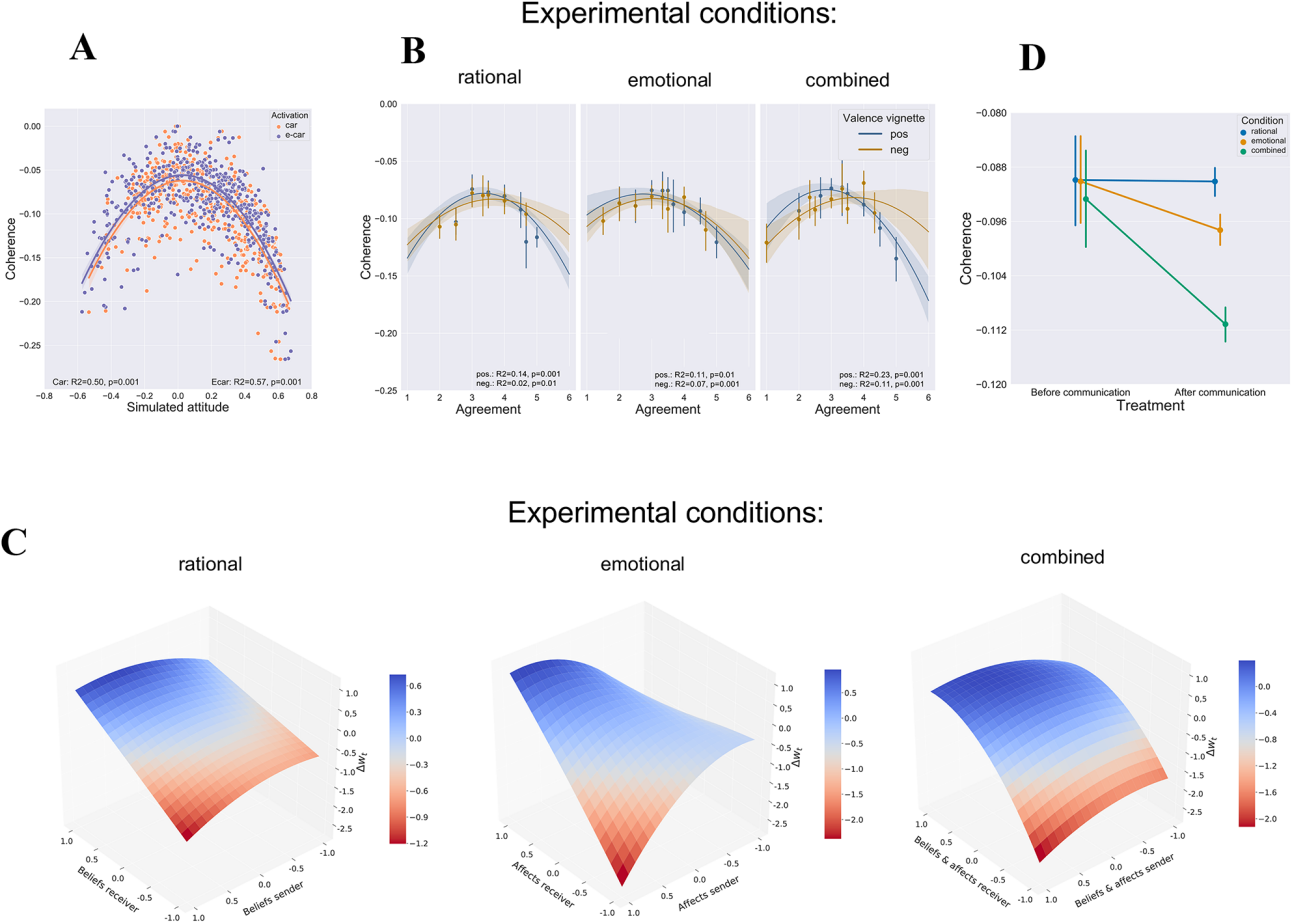
Computational model results. Note in general low levels of energy indicate a high degree of coherence. (**A**) Correlation of simulated attitudes for conventional (orange) and electric cars (blue) with simulated coherence, i.e. level of energy, of PCS network models for all simulated persuasive appeals. The solid lines are the correlation lines and shaded areas indicate a 95% CI. (**B**) Regression of empirical measured agreement to statements for each condition and the corresponding simulated coherence of PCS networks. Solid lines (blue: positively valenced; gold negatively valenced) are the best-fit regression lines. For the sake of better readability of the graph, empirical ratings of agreement are aggregated in bins. The error bars show the 95% CI. (**C**) Average weight changes of beliefs and affect link calculated based the observed attitude changes for each condition. For PCS networks in the rational condition error function *D* is minimized for free parameters *b1 =0; b2 =0.7*,* b3 =−0.3*,* b4 = 0 b5 = −0.3*, in the emotional condition for the parameters *b1 =0; b2 =0.7*,* b3 =−0.3*,* b4 = 0.3 b5 = −0.7* and in the combined condition where *b1 =0; b2 =1.0*,* b3 =-0.3*,* b4 = 0.7 b5 = −0.3.* (**D**) The average coherence level of PCS networks before and after the treatment for each condition.

Using the parameterized PCS models, we conducted a simulation of each respondent’s attitude prior to the influence of persuasive messages. To validate whether our model accurately reflects the empirically observed attitude patterns before persuasion attempts, we correlated the activations of the option nodes for ICE cars and E-cars with the corresponding self-reported attitudes of the respondents. These results demonstrated a strong resemblance between the simulations and the empirical findings (ICE car: *r* = 0.93, *p* = 0.001; E-car: *r* = 0.93, *p* = 0.001). Furthermore, we investigated the relationship between the simulated coherence of networks, quantified by the energy of PCS networks, and the respondents’ simulated prior attitudes across different conditions. Our findings revealed an inverted U-shaped pattern (Fig. 4A), which was also supported by the results of quadratic regression models for ICE cars (*R*^2^ = 0.50, *F*(1, 478) = 476.4, *p* < 0.001) and for E-cars (*R*^2^ = 0.57, *F*(1, 478) = 631.7, *p* < 0.001). As predicted by the PCS approach, both strongly negative and strongly positive prior attitudes were associated with low levels of energy, indicating that these attitudes were consistent, stable, and resistant to change. In Fig. 4B, we examine how empirically measured agreement with statements relates to the coherence levels (energy) of participants’ initial attitude structures, as captured by our PCS network simulations prior to message presentation. The analysis reveals a systematic inverted U-shaped relationship between message agreement ratings and PCS network energy across all experimental conditions, though with varying strengths (rational pos.: *R*^2^ = 0.14, *F*(2, 157) = 13.86, *p* < 0.001; rational neg.: *R*^2^ = 0.02, *F*(2, 157) = 2.69, *p* < 0.01; emotional pos.: *R*^2^ = 0.11, *F*(2, 157) = 11.29, *p* < 0.01; emotional neg.: *R*^2^ = 0.07, *F*(2, 157) = 6.65, *p* < 0.001; combination pos.: *R*^2^ = 0.23, *F*(2, 157) = 24.12, *p* < 0.001; combination neg.: *R*^2^ = 0.11, *F*(2, 157) = 10.96, *p* < 0.001).

This pattern aligns with predictions from PCS theory: strong agreement with messages is associated with low energy levels (high coherence), indicating stable initial attitude structures. While low agreement is also associated with low energy levels, this relationship is less pronounced. In contrast, moderate agreement corresponds to high energy levels (low coherence), reflecting less stable initial attitude structures. The relationship is particularly pronounced for positively valanced messages and strongest in the combined condition, suggesting that messages containing both rational and emotional elements have the most systematic relationship with initial attitude coherence. These findings provide important validation for our PCS modeling approach by demonstrating that the theoretical concept of coherence, as captured by network energy prior to message exposure, systematically relates to subsequent agreement ratings.

To what extent are the cognitive-affective structures that underlie attitudes influenced by persuasive messages with different levels of emotionality? Fig. 4C displays the average weight changes (*∆w*) of beliefs and affect in each experimental condition that minimize the respective error functions *D*, representing the gap between simulated and measured attitudes following persuasive communication. The results reveal diverse patterns of belief and affect convergence and divergence across conditions. These patterns depend on the strength and valence of the prior beliefs and affect held by receivers, as well as their disparity from those of the sender.

In the rational condition, for instance, when the sender and receiver maintain strong opposing prior beliefs (sender: *w*_*s*_ = + 1.0/-1.0 and receiver *w*_*r*_ = − 1.0/+1.0), receivers tend to adjust their beliefs in a direction contrary to the new information, reinforcing the strength of their initial beliefs. However, the extent of this backfire effect differs between receiving agents with prior positive and negative beliefs. Interestingly, in response to opposing positive information, this backfire effect leads to more significant negative weight adjustments than belief changes induced by negative messages that align with pre-existing negative beliefs. Conversely, when the sender supports the receivers’ prior beliefs, the weights are updated in the direction of the sender.

The most pronounced effects of belief polarization/convergence and valence asymmetry are observed when two agents exclusively exchange affect, particularly in the emotional condition and, to a slightly lesser extent, in the combined condition. Intriguingly, in the emotional condition, opposing negative messages (i.e., when the receiver holds strong positive affect) do not lead to polarization but instead prompt a convergence of posterior affect towards the message. In contrast, when the recipient has a negative affect towards the attitude object and receives positive information about it, the backfire effect is most pronounced, with mental structures underlying the attitudes changing more strongly in the opposite direction to the message than in all other conditions. Additionally, contrary to expectations, under a negative constant condition (i.e., both sender and receiver are negative), the weight adjustments shift in a positive direction. These specific convergence and divergence effects underscore the unique role of emotional communication in moderating attitude changes, which can either reduce or increase polarization. To ascertain the impact of weight adjustments in response to persuasive messages on the coherence of PCS networks, we conducted a comparison of the average energy levels among all agents before and after the treatment for each condition (Fig. 4D). Prior to receiving persuasive messages, no significant differences in coherence levels were observed between conditions (*F*(2,477) = 0.19, *p* = 0.8824, η^2^ = 0.001). However, exposure to persuasive information resulted in a statistically significant decrease in energy in the emotional and combined conditions, but not in the rational condition (*F*(1,479) = 410.96, *p* = 0.0001, η^2^ = 0.0462). These findings align with the expectations of the coherence maximization process, indicating that beliefs and affect are adjusted in a manner that enhances the coherence among existing mental representations. Notably, this effect is most pronounced when both rational and emotional communication strategies are employed simultaneously.

## Discussion

Our research had two primary goals. First, we aimed to investigate the impact of different levels of emotional content in persuasive messages on motivated reasoning. Our findings revealed that regardless of message framing, motivated reasoning influenced the persuasiveness of statements, resulting in dissonance with the message and a tendency to discredit the information source, particularly when individuals encountered opposing viewpoints. Moreover, we identified backfire effects, where individuals with strongly held views and high dissonance actually reinforced their existing attitudes, consistent with previous research^59^. A novel aspect of our study was that the extent of this process was moderated by message type and the valence of the advocated positions. Biased reasoning was most prevalent when participants encountered negatively framed appeals and when messages employed a combined rational and emotional tone, rather than exclusively rational or emotional approaches. The persuasiveness of messages was inversely related to the degree of motivated reasoning. Additionally, our results suggested that the impact of motivated reasoning on perceptions of the source was primarily determined by dissonance (agreement) with persuasive appeals, rather than by prior attitudes.

Our second goal was to elucidate the observed effects using computational mechanisms related to emotional coherence, achieved by extending an existing parallel constraint satisfaction (PCS) model^40^ with a learning algorithm. Our simulation results indicated that modifications of the existing mental representations underlying attitude changes exhibited significant variations across valence and different message types. Consistent with prior experiments^60^, we found that negative affect and beliefs were more resistant to counterattitudinal persuasive attempts. Furthermore, we demonstrated that negative mental structures had a greater tendency to lead to backfire effects, which were most pronounced when emotionally positive appeals were encountered. These findings suggested that mental structures respond to and integrate information with varying emotional qualities and intensities. Recent neuroimaging studies supported the idea that positively and negatively framed information is processed differently in the brain and is differentially used to update prior affect and beliefs^61^.

Previous research on PCS models^62^, which assumed coherence maximization as the core mechanism of information integration, argued that this process naturally operates to increase the coherence of existing mental representations. By employing a more realistic learning PCS network model, we complemented these earlier studies by providing evidence that the level of coherence shift associated with attitude changes depended on the type of mental structure involved in information integration. Information that solely influenced the cognitive structures of attitudes resulted in no increase in overall coherence after updating beliefs. In contrast, when information involved affective and, particularly, both affective and cognitive elements of attitudes, global coherence significantly increased. These findings emphasized the role of affect in reducing dissonance and ambivalence toward options for action and supported the notion that decision and attitude formation are driven by maximizing the emotional coherence of mental structures, combining cognition and affect, relevant to the decision at hand^40^. Furthermore, the pattern of coherence shifts, inversely related to the observed tendency of motivated reasoning, suggested an effortful process underpinning the integration of new information, especially when strong cognitive and affective components of attitudes were involved. The greater the effort required for a person to arrive at a coherent conclusion, the lower the persuasiveness and credibility of the message source. Future research could integrate additional elements such as specific identity concepts into the model, further investigating the interaction between the goal of attitude protection and the goal of identity protection, both of which moderate responses to new information.

Our study has some limitations that should be acknowledged. First, the perceived attitude change effects reached statistical significance only after adjusting for perceived dissonance and prior attitudes, which raises questions about the stability and robustness of these findings, particularly given the absence of preregistration. Future research should aim to replicate these effects using preregistered designs to strengthen confidence in the results. Second, we focused on a single climate-relevant issue: vehicle choices. While this approach allowed for a detailed exploration and theoretical explanation of the cognitive-affective mechanisms underlying motivated reasoning in interpersonal communication, it remains unclear whether the empirical results would hold for other topics. Specific characteristics of the issue—such as personal relevance, economic implications, infrastructure dependencies, and public salience—limit the generalizability of our empirical findings to this domain. Furthermore, our study’s participants were exclusively from Germany, posing a limitation on the cross-cultural applicability of our results. To address these constraints, future research could apply the proposed computational model to explore other domains where technological innovations intersect with personal attitudes and societal influence, such as renewable energy adoption or smart technology usage in homes, drawing upon diverse national samples. We posit that the cognitive-affective mechanisms identified through our research may also apply to similar contexts.

Despite these limitations, our findings make a valuable contribution to the growing body of literature on the role of motivated reasoning in information processing and attitude formation. While earlier studies focused on the influence of belief strength, values, political or group identity on attitude responses and group polarization in the context of biased processing of evidence and persuasive appeals^36^, we extended this work by demonstrating that positively valanced and cognitively framed messages can mitigate reasoning bias and prevent polarization between communication partners. Our results suggested that reducing the emotional content of messages that contradict individuals’ beliefs and affect, while increasing emotional intensity in persuasive appeals that align with respondents’ pre-existing beliefs and affective connotations of the attitude object, may offer an effective strategy to enhance the persuasiveness of communication and mitigate backfire effects^63^.

Additionally, our research contributes to the field of computational models of opinion dynamics and social influence^64^. Our findings underscore the importance of studying and comprehending the microfoundations of processes underlying motivated reasoning. Our modeling framework exemplifies how existing theoretical models of attitude and opinion formation can be extended to incorporate psychologically realistic processes related to information processing, with empirical support as a basis. We anticipate that our approach paves the way for future research aimed at understanding how individual reasoning influences dynamics at a population level. This approach has practical implications, such as in policy contexts related to the transition of mobility, where it can enhance the understanding of how policymakers can effectively communicate policy issues, accelerate public comprehension of available evidence, and foster informed policy discussions^65,66^.

## Electronic supplementary material

Below is the link to the electronic supplementary material.

Supplementary Material 1

## References

[1] IPCC: Sections. In: Climate Change 2023: Synthesis Report. Contribution of Working Groups I, II and III to the Sixth Assessment Report of the Intergovernmental Panel on Climate Change. (2023). pp. 35–115. 10.59327/IPCC/AR6-9789291691647 (2023).

[2] Sierzchula, W., Bakker, S., Maat, K. & Van Wee, B. The influence of financial incentives and other socio-economic factors on electric vehicle adoption. *Energy Policy*. **68**, 183–194 (2014).

[3] Chen, Z., Carrel, A. L., Gore, C. & Shi, W. Environmental and economic impact of electric vehicle adoption in the U.S. *Environ. Res. Lett.* **16**, 045011 (2021).

[4] Singh, V., Singh, V. & Vaibhav, S. A review and simple meta-analysis of factors influencing adoption of electric vehicles. *Transp. Res. D Transp. Environ.* **86**, 102436 (2020).

[5] Egbue, O., Long, S. & Samaranayake, V. A. Mass deployment of sustainable transportation: evaluation of factors that influence electric vehicle adoption. *Clean. Technol. Environ. Policy*. 10.1007/s10098-017-1375-4 (2017).

[6] Zimm, C. Improving the understanding of electric vehicle technology and policy diffusion across countries. *Transp. Policy*. **105**, 54–66 (2021).

[7] Wolske, K. S., Gillingham, K. T. & Schultz, P. W. peer influence on household energy behaviours. *Nat. Energy*. **5**, 202–212 (2020).

[8] Pettifor, H., Wilson, C., Axsen, J., Abrahamse, W. & Anable, J. Social influence in the global diffusion of alternative fuel vehicles – A meta-analysis. *J. Transp. Geogr.* **62**, 247–261 (2017).

[9] Petty, R. E. & Cacioppo, J. T. The Elaboration Likelihood Model of Persuasion. *Adv. Exp. Soc. Psychol.* **19**, 123–205 (1986).

[10] McGuire, W. Constructing social psychology. *Constr. Soc. Psychol.*10.1017/CBO9780511571206 (1999).

[11] Chen, S. & Chaiken, S.* The heuristic-systematic model in its broader context.* In Dual-process Theories in Social Psychology (eds Chaiken, S. & Trope, Y.) The Guilford Press, 73–96. (1999).

[12] Jost, J. T., Baldassarri, D. S. & Druckman, J. N. Cognitive–motivational mechanisms of political polarization in social-communicative contexts. *Nat, Rev. Psychol.* **1**, 560–576. (2022). 10.1038/s44159-022-00093-5 PMC9342595 35937553

[13] Druckman, J. N. A Framework for the study of Persuasion. *Annu. Rev. Polit. Sci.* **25**, 65–88 (2022).

[14] Kunda, Z. The case for motivated reasoning. *Psychol. Bull.* **108**, 480–498 (1990). 2270237 10.1037/0033-2909.108.3.480

[15] Westen, D., Blagov, P. S., Harenski, K., Kilts, C. & Hamann, S. Neural bases of motivated reasoning: an fMRI study of emotional constraints on partisan Political Judgment in the 2004 U.S. Presidential Election. *J. Cogn. Neurosci.* **18**, 1947–1958 (2006). 17069484 10.1162/jocn.2006.18.11.1947

[16] Mathews, A. & Mackintosh, B. Induced emotional interpretation bias and anxiety. *J. Abnorm. Psychol.* **109**, 602–615 (2000). 11195984

[17] Munro, G. D., Ditto, P. H. & Biased Assimilation Attitude polarization, and affect in reactions to stereotype-relevant Scientific Information. *Pers. Soc. Psycol. Bull.* **23**, 636–653. 10.1177/0146167297236007 (1997).

[18] Everaert, J., Duyck, W. & Koster, E. H. W. Attention, interpretation, and memory biases in subclinical depression: a proof-of-principle test of the combined cognitive biases hypothesis. *Emotion* **14**, 331–340 (2014). 24512247 10.1037/a0035250

[19] Ma, Y., Dixon, G. & Hmielowski, J. D. Psychological reactance from reading Basic facts on Climate Change: the role of prior views and political identification. *Environ. Commun.* **13**, 71–86 (2019).

[20] Koehler, J. J. The Influence of Prior Beliefs on Scientific Judgments of Evidence Quality. *Organ. Behav. Hum. Decis. Process.*10.1006/obhd.1993.1044 (1993).

[21] Braman, E. & Nelson, T. E. Mechanism of motivated reasoning? Analogical perception in discrimination disputes. *Am. J. Pol. Sci.* **51**, 940–956 (2007).

[22] Wolsko, C., Ariceaga, H. & Seiden, J. Red, white, and blue enough to be green: effects of moral framing on climate change attitudes and conservation behaviors. *J. Exp. Soc. Psychol.* **65**, 7–19 (2016).

[23] Kahan, D. M. et al. The polarizing impact of science literacy and numeracy on perceived climate change risks. *Nat. Clim. Chang.* **2**, 732–735 (2012).

[24] Bayes, R., Druckman, J. N., Goods, A. & Molden, D. C. When and how different motives can drive motivated political reasoning. *Polit Psychol.* **41**, 1031–1052 (2020).

[25] Ecklund, E. H., Scheitle, C. P., Peifer, J. & Bolger, D. Examining links between Religion, evolution views, and Climate Change Skepticism. *Environ. Behav.* **49**, 985–1006 (2017).

[26] Jacks, J. Z., Cameron, K. A., Jacks, J. Z. & Cameron, K. A. Strategies for resisting persuasion. *Basic. Appl. Soc. Psych*. **25**, 145–161 (2003).

[27] Bail, C. et al. Exposure to opposing views can increase political polarization: evidence from a large-scale field experiment on social media. *Proc. Natl. Acad. Sci.*. **117**, 9216–9221 (2018). 10.1073/pnas.1804840115 PMC6140520 30154168

[28] Druckman, J. N., Peterson, E. & Slothuus, R. How elite partisan polarization affects public opinion formation. *Am. Polit. Sci. Rev.* **107**, 57–79 (2013).

[29] Merkley, E. & Stecula, D. A. Party cues in the news: democratic elites, republican backlash, and the dynamics of Climate Skepticism. *Br. J. Polit. Sci.* **51**, 1439–1456 (2021).

[30] Swire-Thompson, B., DeGutis, J. & Lazer, D. Searching for the Backfire Effect: measurement and design considerations. *J. Appl. Res. Mem. Cogn.* **9**, 286–299 (2020). 32905023 10.1016/j.jarmac.2020.06.006 PMC7462781

[31] Hutmacher, F., Reichardt, R. & Appel, M. Motivated reasoning about climate change and the influence of Numeracy, need for Cognition, and the Dark factor of personality. *Sci. Rep.*10.1038/s41598-024-55930-9 (2024). 38454097 10.1038/s41598-024-55930-9 PMC10920913

[32] Fischer, H., Huff, M. & Said, N. Polarized climate change beliefs: no evidence for science literacy driving motivated reasoning in a U.S. national study. *Am. Psychol.* **77**, 822–835 (2022). 35467910 10.1037/amp0000982

[33] Hart, P. S., Nisbet, E. C. & Myers, T. A. Public attention to science and political news and support for climate change mitigation. *Nat. Clim. Change* **5**, 541–545. (2015).

[34] Stoetzer, L. S. & Zimmermann, F. A representative survey experiment of motivated climate change denial. *Nat. Clim. Change*** 14**, 198–204. (2024).

[35] Bayes, R. & Druckman, J. N. Motivated reasoning and climate change. *Curr. Opin. Behav. Sci.* **42**, 27–35 (2021).

[36] Druckman, J. N. & McGrath, M. C. The evidence for motivated reasoning in climate change preference formation. *Nat. Clim. Change*. **9**, 111–119 (2019).

[37] Bago, B., Rand, D. G. & Pennycook, G. Reasoning about climate change. *PNAS Nexus* **2**, pgad100. (2023). 10.1093/pnasnexus/pgad100 PMC10153421 37143867

[38] Melnikoff, D. E. & Strohminger, N. Bayesianism and wishful thinking are compatible. *Nat. Hum. Behav. 2024*. **8**(4), 692–701. (2024). 10.1038/s41562-024-01819-6 38396212

[39] Thagard, P. *Coherence in Thought and Action* MIT Press. (2000).

[40] Thagard, P. *Hot Thought: Mechanisms and Applications of Emotional Cognition* MIT Press. (2006).

[41] Simon, D. & Read, S. J. Toward a General Framework of biased reasoning: coherence-based reasoning. *Perspect. Psychol. Sci.*10.1177/17456916231204579 (2023). 37983541 10.1177/17456916231204579

[42] Monroe, B. M. & Read, S. J. A general connectionist model of attitude structure and change: the ACS (attitudes as constraint satisfaction) model. *Psychol. Rev.* **115**, 733–759 (2008). 18729597 10.1037/0033-295X.115.3.733

[43] Thagard, P. & Verbeurgt, K. Coherence as constraint satisfaction. *Cogn. Sci.* **22**, 1–24 (1998).

[44] Read, S. J. & Simon, D.* Parallel constraint satisfaction as a mechanism for cognitive consistency.* In Cognitive Consistency: A Fundamental Principle in Social Cognition. Guilford Press, 66–86. (2012).

[45] Glöckner, A. & Betsch, T. Decisions beyond boundaries: when more information is processed faster than less. *Acta Psychol.*. **139**, 532–542 (2012). 22381940 10.1016/j.actpsy.2012.01.009

[46] Jekel, M., Glöckner, A. & Bröder, A. A new and unique prediction for cue-search in a parallel-constraint satisfaction network model: the attraction search effect. *Psychol. Rev.* **125**, 744–768 (2018). 29952587 10.1037/rev0000107

[47] Kunda, Z. & Thagard, P. Forming impressions from stereotypes, traits, and behaviors: a parallel- constraint- satisfaction theory. *Psychol. Rev.* **103**, 284–308 (1996).

[48] Heck, D. W. & Erdfelder, E. Linking process and measurement models of recognition-based decisions. *Psychol. Rev.* **124**, 442–471 (2017). 28368144 10.1037/rev0000063

[49] Festinger, L. *The Theory of Cognitive Dissonance* (Stanford University Press, 1957).

[50] Osgood, C. E. & Tannenbaum, P. H. The principle of congruity in the prediction of attitude change. *Psychol. Rev.* **62**, 42–55 (1955). 14357526 10.1037/h0048153

[51] Simon, D., Stenstrom, D. M. & Read, S. J. The coherence effect: blending cold and hot cognitions. *J. Pers. Soc. Psychol.* **109**, 369–394 (2015). 26167800 10.1037/pspa0000029

[52] Taber, C. S. & Lodge, M. Motivated skepticism in the evaluation of political beliefs. *Am. J. Pol. Sci.* **50**, 755–769 (2006).

[53] Nordmo, M. & Selart, M. The asymmetrical force of persuasive knowledge across the positive–negative divide. *Front. Psychol.* **6**, 1324 (2015). 26388821 10.3389/fpsyg.2015.01324 PMC4559650

[54] Unkelbach, C., Fiedler, K., Bayer, M., Stegmüller, M. & Danner, D. Why positive information is processed faster: the density hypothesis. *J. Pers. Soc. Psychol.* **95**, 36–49 (2008). 18605850 10.1037/0022-3514.95.1.36

[55] McClelland, J. L. & Rumelhart, D. E. An interactive activation model of context effects in letter perception: part I. An account of basic findings. *Psychol. Rev.* **88**, 365–405 (1981). 7058229

[56] Rescorla, R. A. & Wagner, A. R. A theory of pavlovian conditioning: variations in the effec- tiveness of reinforcement and nonreinforcement. in Classical Conditioning II: Current Research and Theory (eds Black, A. H. & Prokasy, W. F.). Appleton-Century-Crofts, 65–99. (1972).

[57] Read, S. J. & Urada, D. I. A neural network simulation of the outgroup homogeneity effect. *Pers. Soc. Psychol. Rev.* **7**, 146–169 (2003). 10.1207/S15327957PSPR0702_146-169 12676645

[58] Read, S. J. & Monroe, B. M. Modeling cognitive dissonance as a parallel constraint satisfaction network with learning. *Cognitive Dissonance: Reexamining a Pivotal Theory in Psychology (2nd ed.)* 197–226. (2019). 10.1037/0000135-010

[59] Nyhan, B. & Reifler, J. When corrections fail: the persistence of political misperceptions. *Polit. Behav.* **32**, 303–330 (2010).

[60] Bizer, G. Y. & Petty, R. E. How we conceptualize our attitudes matters: the effects of valence framing on the resistance of political attitudes. *Polit. Psychol.* **26**, 553–568 (2005).

[61] Sharot, T. & Garrett, N. Forming beliefs: why Valence matters. *Trends Cogn. Sci.* **20**, 25–33 (2016). 26704856 10.1016/j.tics.2015.11.002

[62] Glöckner, A. & Betsch, T. Modeling option and strategy choices with connectionist networks: towards an integrative model of automatic and deliberate decision making. *Judgm. Decis. Mak.* **3**, 215–228 (2008).

[63] Wolf, I. & Schröder, T. Connotative meanings of sustainable mobility: a segmentation approach using cultural sentiments. *Transp. Res. Part. Policy Pract.* **126**, 259–280 (2019).

[64] Flache, A. et al. Models of Social Influence: towards the next frontiers. *J. Artif. Soc. Soc. Simul.* **20**, 2 (2017).

[65] Wolf, I., Schröder, T., Neumann, J. & de Haan, G. Changing minds about electric cars: an empirically grounded agent-based modeling approach. *Technol. Forecast. Soc. Change*. **94**, 269–285 (2015).

[66] Schröder, T. & Wolf, I. Modeling multi-level mechanisms of environmental attitudes and behaviours: the example of carsharing in Berlin. *J. Environ. Psychol.* **52**, 136–148 (2017).

