# Supplementary material for: The critical role of emotional communication for motivated reasoning
Source: Sci Rep. 2024 Dec 30;14:31681. doi: 10.1038/s41598-024-81605-6 (PMC11685395; doi:10.1038/s41598-024-81605-6)
Supplement: Supplementary file 1 — Supplementary Material 1 [file 41598_2024_81605_MOESM1_ESM.pdf]

Supplementary information to the manuscript "The critical role of emotional communication for motivated reasoning"

Authors

Ingo Wolf*
Research Institute for Sustainability – Helmholtz Centre Potsdam (RIFS), Potsdam, Germany

Tobias Schröder
Potsdam University of Applied Sciences, Potsdam
Kiepenheuerallee 5
14469 Potsdam, Germany

Corresponding Author

* Ingo Wolf
Research Institute for Sustainability – Helmholtz Centre Potsdam (RIFS)
Berliner Straße 130
14467 Potsdam, Germany
Email: ingolupo@gmx.de

Table A1. Vignette dimensions and characteristics.

Supplementary Table A1 displays all vignette dimensions (factors) and their respective levels (characteristics).

Dimension/factors	***Levels/characteristics***	***Examples of levels***
Type of persuasive communication	1. Rational<br>2. Emotional<br>3. Combination of emotional and rational	1. “When choosing a suitable means of transportation in my daily life, it is especially important to me …”<br>2. “Driving a combustion engine car is just pure fun!”<br>3. “Driving a combustion engine car is just pure fun! Moreover, it meets my needs like no other means of transportation when it comes to ...”
Type of vehicle powertrain	1. Internal combusting engine car<br>2. Electric engine car	1. “… driving a combustion engine car …”<br>2. “… driving an electric car …”
Beliefs about contribution to domain-specific goals/needs	1. Independence<br>2. Comfort<br>3. Eco-friendliness<br>4. Driving experience<br>5. Guilt conscience<br><br>Note: two characteristics per vignette	1. “A combustion engine car provides a high degree of independence, allowing me to take control of my life to some extent.” [rational condition, ICE, positive valence]<br>2. “Inferior materials, poor craftsmanship, and seats as comfortable as park benches. That’s zero relaxation! I think it's an outrage!“ [emotional condition, EV, negative valence]<br>3. “I really don’t get what everyone is always going on about: fuel-efficient driving or, even better, giving up cars altogether – nonsense. There are far worse environmental offenders than combustion engine cars, that’s for sure.“ [combined condition, ICE, positive valence]<br>4. “Nowadays, combustion engine cars are equipped with so many assistance systems that the driving experience takes a back seat.” [rational condition, ICE, negative valence]<br>5. “I no longer need to feel guilty when I get into my electric car: I’m doing something for the future of our children, and I think that’s fantastic!“ [emotional condition, EV, positive valence]
Valence of attitude object	1. Positive<br>2. Negative	1. “Overall, the combustion engine cars meet all my requirements, and I can only recommend using it.“ [rational condition, ICE]<br>2. “I certainly won't be using an electric car, and I can only recommend that you do the same. [combined condition, EV]

Table A2: Vignette examples for the experimental conditions rational, emotional and combined.

Supplementary Table A2 displays the English translation of vignette examples for the three experimental conditions, i.e. rational persuasion, emotional persuasion, and the combined persuasion condition, in which vignettes argue for both goal-oriented and emotional aspects.

Condition	***Vignette example***	***Factor levels***
Rational persuasion	When I decide on a suitable means of transport for everyday life, it is important to me that I can travel comfortably and in an environmentally friendly way. The car satisfies my need for environmentally friendly mobility, as I can also drive in an environmentally friendly way if I drive it in the right way. In addition, today's cars offer me great comfort due to their modern equipment. All in all, the car meets all my requirements and I can only recommend that you drive it.	- *vehicle powertrain*: internal combustion engine<br>- *domain-specific goals*: environmental friendliness, comfort<br>- *valence of attitude object*: positive
Emotional persuasion	Electric cars are absurd! I think it's outrageous—they use subpar materials, the craftsmanship is poor, and the seats might as well be park benches. There's absolutely no comfort! The whole concept of electro mobility seems like self-deception. How does it contribute to environmental protection if someone buys an electric car as a third vehicle? This is definitely not the solution to our environmental issues.	- *vehicle powertrain*: electric engine/car<br>- *domain-specific goals*: environmental friendliness, comfort<br>- *valence of attitude object*: negative
Rational and emotional persuasion combinded	Driving a car is just fun! It satisfies my need for comfort like no other means of transport and is for me an environmentally friendly form of mobility. After work, get in the car and come down first. In the comfortable ambience of my car. I can always relax wonderfully. I don't even know what they all have: Fuel-efficient driving or even better, completely doing without the car, nonsense. God knows there are worse environmental sinners than car drivers. For me, the car is the perfect means of transport, and I think it's the best for you too!	- *vehicle powertrain*: internal combustion engine<br>- *domain-specific goals*: environmental friendliness, comfort<br>- *valence of attitude object*: positive
